# Complete mitochondrial genome of the Chihuil sea catfish *Bagre panamensis* (Siluriformes: Ariidae)

**DOI:** 10.1080/23802359.2017.1334519

**Published:** 2017-06-01

**Authors:** Jorge S. Ramírez-Pérez, Nancy C. Saavedra-Sotelo, Raúl Llera-Herrera, Quetzalli Yasu Abadía-Chanona

**Affiliations:** a Facultad de Ciencias del Mar, Universidad Autónoma de Sinaloa, Mazatlán, Mexico;; b Facultad de Ciencias del Mar, CONACYT-Universidad Autónoma de Sinaloa, Mazatlán, Mexico;; c Unidad Acuicultura y Manejo Ambiental, CONACYT-Centro de Investigación en Alimentación y Desarrollo A. C, Mazatlán, Sinaloa, Mexico

**Keywords:** Sea catfish, Bagre panamensis, mitogenome, Illumina

## Abstract

The chihuil sea catfish (*Bagre panamensis*) is endemic of the Eastern Pacific and is a species of fishery importance in the Mexican Pacific. The complete mitochondrial genome of *Bagre panamensis* has been assembled from Illumina sequencing data. The circular genome was 16,714 bp in lengh, and consist of 13 protein-coding, two ribosomal RNAs (rRNAs), and 22 transfer RNA (tRNA) genes. Base composition is 30.8% A, 26.6% T, 28.2% C, and 14.4% G, and 42.6% GC content. Protein-coding genes present two start codon (ATG and GTG) and eight stop codon (TAA, TCT, CCT, TTA, CAT, AAT, ATT, and TAG). The control region possesses the highest A + T (64.4%) and lowest G + C content (35.6%) among all mitochondrial regions. These data would contribute to the evolutionary studies of related taxa.

The Ariidae familie (Order: Siluriformes) is widely distributed in world tropical shelves, includes 150–200 species approx. of which one third is endemic to American coasts (Betancur-R et al. 2007; Marceniuk & Menezes 2007). The Ariidae familie present a monophyletic ancestry, well-supported by morphological and molecular traits (Diogo 2004; Kailola 2004; Sullivan et al. 2006; Betancur-R et al. 2007); however, the systematics of its species is complex and there are many problems on its nomenclatural (Marceniuk & Menezes 2007). The ariids are predominantly marine species of which the chihuil catfish (*Bagre panamensis*) is endemic of the Eastern Pacific, it is distributed from southern California to northern Peru including Gulf of California and Galapagos Islands (Cooke 1992; Allen & Robertson 1994). *Bagre panamensis* is a demersal fish that habitat in muddy bottoms near to shore (177 m depth approx.), estuaries and mangroves (Cooke 1992).

In this study, we determined the complete mitochondrial genome of *B. panamensis* for first time. One specimen was collected from artisanal fishery Sinaloa, Mexico (23°28′32.5″N - 106°37′28.2″W). DNA was extracted from fresh muscle tissue using the Wizard® Genomic DNA Purification kit (Promega, Madison, WI). A genomic DNA library was constructed with the Kapa DNA library preparation kit (Kapa Biosystems, Wilmington, MA) using multiplex index, and the library was then sequenced alongside other barcoded libraries using a single lane (2 × 125 paired-end reads) in a MiSeq platform (Illumina, San Diego, CA). Reads were pre-processed using Trimmomatic v0.33 (Bolger et al. 2014) for trim low-quality ends (Q score <20), residual adapters and remove reads shorter than 100 bases. The obtained sequences were demultiplexed, and the recovered reads were analysed for quality control with FastQC v0.10.1 (Babraham Institute, Cambridge, UK) (Andrews 2011). 21′391,977 pair of high-quality reads (Q score >25) were recovered. The complete mitochondrion genome was obtained using MITObim v1.7 (Hahn et al. 2013), using the giant catfish mitogenome *Netuma thalassina* (GenBank accession number: KU986659.1) as a reference. The final assembly was annotated using MitoAnnotator pipeline (Iwasaki et al. 2013).

The mitogenome of *B. panamensis* (GenBank accession number KY930718) has a length of 16,673 bp with a base composition of A 38.8%, T 26.6%, C 28.2%, and G 14.4% (42.6% of GC content). The mitogenome contains all typical genes of vertebrate: 13 protein-coding genes, 22 transference RNA genes, two ribosomal RNAs, and one control region or *d-loop* (Figure 1). Almost all protein-coding genes initiated by typical ATG codon, except for the *COX1* gene initiated by the GTC. For the stop codon, almost all genes presented TAA or CCT, the rest used a different one (Table 1). The control region (*D-loop*) is 1080 bp in length which was located between the *tRNA-Pro* and *tRNA-Phe*; it had the highest A + T content of 64.4% and lowest G + C content (35%) among all mitochondrial regions.

**Figure 1.**
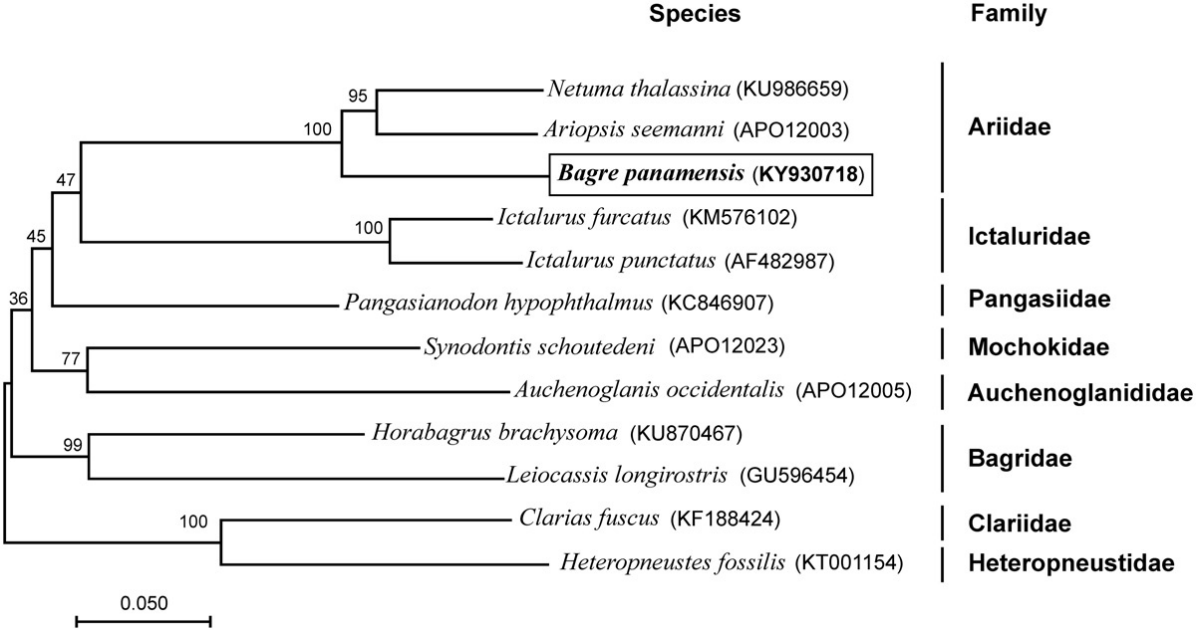
Maximum-likelihood (ML) phylogenetic tree of *Bagre panamensis* and the other 11 species of 8 families using *Clarias fuscus* and *Heteropneustes fossilis* as an outgroup. Number above each node indicates the ML bootstrap support values. In parenthesis the access numbers from NCBI database.

**Table 1.** Annotation of the complete mitochondrial genome of *Bagre panamensis*.

Gene name	Location (bp)	Length (bp)	Start codon	Stop codon
*tRNA-Phe*	1–70	70		
*12SrRNA*	71–1030	960		
*tRNA-Val*	1031–1102	72		
*16SrRNA*	1103–2778	1676		
*tRNA-Leu*	2779–2853	75		
*NAD1*	2854–3825	972	ATG	TAA
*tRNA-Ile*	3826–3897	72		
*tRNA-Gln*	3898–3968	71		
*tRNA-Met*	3969–4038	70		
*NAD2*	4039–5080	1042	ATG	TCT
*tRNA-Trp*	5081–5153	73		
*tRNA-Ala*	5154–5222	69		
*tRNA-Asn*	5223–5295	73		
*tRNA-Cys*	5296–5362	67		
*tRNA-Tyr*	5363–5432	70		
*COX1*	5433–6983	1551	GTG	TAA
*tRNA-Ser*	6984–7054	71		
*tRNA-Asp*	7055–7123	69		
*COX2*	7124–7814	691	ATG	CCT
*tRNA-Lys*	7815–7888	74		
*ATP8*	7889–8056	168	ATG	TAA
*ATP6*	8057–8739	683	ATG	TTA
*COX3*	8740–9523	784	ATG	CAT
*tRNA-Gly*	9524–9596	73		
*NAD3*	9597–9945	349	ATG	AAT
*tRNA-Arg*	9946–10,016	71		
*NAD4L*	10,017–10,313	297	ATG	TAA
*NAD4*	10,314–11,694	1381	ATG	ATT
*tRNA-His*	11,695–11,764	70		
*tRNA-Ser*	11,765–11,831	67		
*tRNA-Leu*	11,832–11,904	73		
*NAD5*	11,905–13,731	1827	ATG	TAA
*NAD6*	13,732–14,244	513	ATG	TAG
*tRNA-Glu*	14,245–14,313	69		
*CYTB*	14,314–15,451	1138	ATG	CCT
*tRNA-Thr*	15,452–15,523	72		
*tRNA-Pro*	15,524–15,593	70		
*D-loop*	15,594–16,673	1080		

To validate the phylogenetic position of *B. panamensis*, we used MEGA6 (Tamura et al. 2013) to construct a maximum-likelihood tree (500 boostrap replicates) containing complete mtDNA of the other 11 species (Figure 1). The phylogenetic position of *B. panamensis* was closely clustered with *Netuma thalassina* and *Ariopsis seemanni*, the three species belong to Ariidae family which are considered new world catfishes.
